# Perioperative posterior reversible encephalopathy syndrome in a patient with no history of hypertension: a case report

**DOI:** 10.1186/s40981-016-0065-2

**Published:** 2016-11-21

**Authors:** Nobuo Sato, Haruhiko Machida, Mitsuharu Kodaka, Keiko Nishiyama, Makiko Komori

**Affiliations:** 1 Department of Anesthesiology, Tokyo Women’s Medical University Medical Center East, 2-1-10, Nishi-ogu, Arakawa-ku, Tokyo, 116-8567 Japan; 2 Department of Radiology, Tokyo Women’s Medical University Medical Center East, Tokyo, Japan; 3 Present address: Department of Anesthesiology, Tokyo Women’s Medical University, 8-1, Kawada-cho, Shinjuku-ku, Tokyo, 162-8666 Japan

**Keywords:** Posterior reversible encephalopathy syndrome, Total hysterectomy, Hypertension, Postoperative pain management, Unconsciousness, Hypoxemia

## Abstract

**Background:**

Posterior reversible encephalopathy syndrome is characterized by reversible neurological symptoms with leukoencephalopathy detectable by computed tomography (CT) and magnetic resonance (MR) imaging.

**Case presentation:**

We here present a patient with no history of hypertension who, after being transferred back to the ward after undergoing total hysterectomy under general anesthesia, had several seizures and lost consciousness. Posterior reversible encephalopathy syndrome was suspected on the basis of brain CT images and clinical findings. She was treated with respiratory support, sedative drugs, and anticonvulsants, and MR imaging confirmed a diagnosis of posterior reversible encephalopathy syndrome. She regained consciousness and responsiveness the following day.

**Conclusions:**

Clinically, posterior reversible encephalopathy syndrome resembles cerebral infarction or intracranial hemorrhage; MR imaging is useful for differentiating it from these conditions. Including this condition in the differential diagnosis and instituting appropriate treatment is important in minimizing the risk of development of irreversible neurological damage during the perioperative period.

## Background

Posterior reversible encephalopathy syndrome (PRES) is a neurological disorder with unique brain computed tomographic (CT) and magnetic resonance (MR) imaging findings [1]. A group of diseases with similar clinical courses and neuro-radiological findings, PRES is characterized by headache, altered mental functioning, seizures, loss of vision, and, typically, hypertension [1, 2]. Reports of PRES have been published increasingly frequently in recent years; however, there are few reports of PRES associated with general anesthesia [3–7], the syndrome having various causes.

We here describe a patient with no history of hypertension who progressively lost consciousness and had several seizures after general anesthesia. She recovered with appropriate treatment in the intensive care unit (ICU) after the diagnosis of PRES had been made from clinical and MRI findings.

## Case presentation

A 46-year-old woman presented to our hospital because of general fatigue. Blood counts showed severe anemia (serum hemoglobin = 2.8 g/dL) and pelvic MR imaging revealed multiple uterine leiomyomas. Otherwise, her physical examination, laboratory tests including blood electrolytes, renal and liver function tests, pulmonary function tests, chest radiographs, electrocardiogram, and transthoracic echocardiography were within normal limits. Her anemia was treated with ferritin and her hemoglobin increased to 11.0 g/dL within 2 months. Total hysterectomy under general anesthesia was scheduled for her uterine leiomyomas. On admission, her body weight was 61 kg, height 1.54 m, blood pressure (BP) 106/64 mmHg, and heart rate 78 beats/minute. She had no history of hypertension.

Prior to surgery, she received 0.5 mg of atropine and 2 mg of midazolam intramuscularly. In the operation room, she was monitored by electrocardiography, noninvasive arterial BP measurements, and a pulse oximeter. Her BP was 161/76 mmHg and heart rate 75 beats/minute before induction of general anesthesia, which was induced with propofol, remifentanil, and rocuronium. Following tracheal intubation, she was ventilated with 50% oxygen and 50% nitrous oxide and sevoflurane, and intermittent doses of fentanyl (total 0.2 mg) were administered intravenously. The operation was uneventful, lasting 1 h and 56 min. Postoperatively, she regained full consciousness and complained of wound pain after extubation. Postoperatively, pain control was achieved with intravenous fentanyl (total 0.2 mg), and she was transferred to the ward.

After her transfer to the ward, 30 mg of pentazocine hydrochloride and 50 mg of hydroxyzine pamoate were administered intravenously in response to further complaints of wound pain. Her BP ranged between 146/84 and 166/100 mmHg in the ward. Two hours after she had been transferred to the ward, she became less responsive; 4 h later she had six episodes of tonic-clonic seizures lasting from 30 s to 2 min and lost consciousness. She was given 5 mg of diazepam intravenously; this terminated the seizures. Even though she was receiving 10 L/minute oxygen by face mask her oxygen saturation remained low at 90%, she was therefore admitted to the ICU. On admission to the ICU, her Glasgow coma scale was 6/15 (motor 3, eyes 2, verbal 1). Her respiration was labored with a rate of 18 per minute and stridor, suggesting upper airway obstruction. Her BP was 112/60 mmHg, heart rate 102 beats/minute, body temperature 36.7°C, and her pupils were 2.5 mm in diameter, equal, and reactive. Initial arterial blood gas analysis while receiving 12 L/minute oxygen administered by a face mask showed a pH of 7.19, PaCO_2_ of 46 mmHg, PaO_2_ of 125 mmHg, HCO_3_^−^ of 17.6 mmol/L, and standard base excess of −10.6 mmol/L. A chest radiograph showed diffuse, slightly increased opacity in both lungs. Brain CT images taken an hour after the seizures occurred showed symmetrical, slightly hypodense areas predominantly in the subcortical white matter of both occipital lobes.

**Fig. 1:**
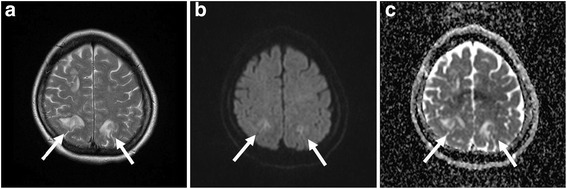
T2-weighted (**a**) and diffusion-weighted (**b**) magnetic resonance images 10 hours after presentation show increased signal intensity in both parietooccipital lobes (*arrows*). The lesions also show increased signal intensity on apparent diffusion coefficient map images (*arrows*) (**c**)

**Fig. 2:**
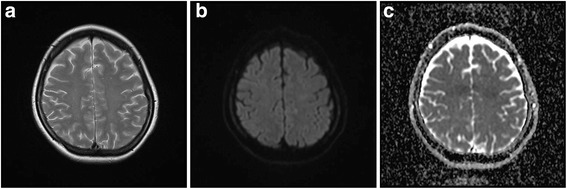
Magnetic resonance imaging study 7 weeks postoperatively shows resolution of all the lesions in T2-weighted (**a**), diffusion-weighted (**b**), and apparent diffusion coefficient map images (**c**)

Based on the aforementioned clinical features and neuroradiological findings, PRES was suspected. The patient was intubated and received mechanical ventilator support while being sedated with midazolam and fentanyl. By 20 min after intubation, her acidosis had improved dramatically, her arterial blood pH being 7.43. To prevent seizures, 250 mg of phenytoin and 800 mg of valproate per day were administered. Phenytoin was administered until the fourth and valproate until the 20th day after the event. On the day after admission to the ICU, the PaO_2_/FiO_2_ ratio was over 500. On brain MR imaging performed 15 h after the seizures had occurred, T2- and diffusion-weighted images revealed symmetrical, increased signal intensity in both parietooccipital lobes (Fig. 1a, b). The lesions showed increased signal intensity on apparent diffusion coefficient map images (Fig. 1c). MR angiography was not suggestive of atherosclerotic changes. After stopping sedation, she regained consciousness and was extubated. She was found to have no neurological deficit except for visual disturbance with hand motion. Her vision had recovered fully by the fourth postoperative day and she was transferred to the ward on the sixth postoperative day. All abnormal findings on brain MR imaging had completely resolved 7 weeks after surgery (Fig. 2a, b and c).

## Discussion

In 1996, the term PRES was proposed for reversible posterior leukoencephalopathy syndrome (RPLS) [1]. The most common causes of PRES/RPLS are hypertensive encephalopathy, preeclampsia/eclampsia, cyclosporin A neurotoxicity, and uremic encephalopathy [1]. Most patients are markedly hypertensive on presentation; however, some have only mildly increased or even normal blood pressure [8, 9]. In PRES/RPLS, T2-weighted MR imaging characteristically shows hyperintensity spreading out from the parietooccipital regions [1, 2]. In the acute phase, diffusion-weighted MR imaging can differentiate ischemic injury from conditions known to cause vasogenic brain edema [9–14].

Alterations in hemodynamic state can lead to PRES. Several reports on PRES associated with general anesthesia have been published [3–7]. Although there are various causes for this syndrome, affected patients typically have hypertension at the time of presentation or a history of hypertension.

In this case, the risk factor for PRES was the sustained hypertension caused by incomplete postoperative pain control [15]. Adequate pain control may have prevented the development of PRES. To prevent PRES, all surgery patients undergoing general anesthesia, including low risk patients undergoing low risk surgery, should be admitted to a postanesthetic care unit for frequent checking of vital signs and to receive adequate postoperative pain control [16].

This clinical report underscores the need to check vital signs carefully to ensure early detection of loss of consciousness that could lead to PRES, even when there is no history of hypertension.

The acquisition of MR images was very useful for the early diagnosis of PRES in this case. Brain MRI is much more sensitive than CT [17]; however CT is sometimes preferable for diagnosing PRES in critically ill patients. The combination of the patient’s clinical features and MR imaging findings led to early and successful treatment.

## Conclusion

We here report the rare complication of delayed postoperative hypertensive encephalopathy. The diagnosis of PRES was made early and appropriate intensive care management of the patient’s symptoms instituted, resulting in complete recovery with no complications.

## Abbreviations

BP: Blood pressure
CT: Computed tomography
ICU: Intensive Care Unit
MR: Magnetic resonance
PRES: Posterior reversible encephalopathy syndrome
RPLS: Reversible posterior leukoencephalopathy syndrome
